# COVID-19-Associated Cystitis: De Novo Urinary Urgency Following SARS-CoV-2

**DOI:** 10.7759/cureus.99386

**Published:** 2025-12-16

**Authors:** Trevor Virno, Nate Tomilonus, Joel Hart, Carl Van Rensburg, Douglas Walsh

**Affiliations:** 1 Medicine, Lake Erie College of Osteopathic Medicine, Bradenton, USA; 2 Internal Medicine, MCR Health, Palmetto, USA

**Keywords:** acute cystitis, coronavirus disease 2019 (covid-19), lower urinary tract symptoms, overactive bladder, urge incontinence

## Abstract

COVID-19 is widely recognized as a systemic disease with pulmonary, cardiovascular, and neurologic manifestations, yet growing evidence highlights the genitourinary tract as another important site of involvement. A distinct clinical entity, COVID-associated-cystitis (CAC), has been described in which patients develop *de novo* or worsening lower urinary tract symptoms (LUTS), such as urgency, frequency, and nocturia, without bacterial infection. This case report describes a 71-year-old woman who developed persistent urinary urgency following recovery from COVID-19, successfully treated with oxybutynin. The case is contextualized within the expanding literature on CAC, including evidence from biomarker studies, mechanistic analyses, and large cohort investigations. Emerging data support a pathophysiologic role for both inflammatory cytokine release and viral receptor expression in the bladder, with symptom overlap between CAC, overactive bladder (OAB), and interstitial cystitis. Recognition of CAC is clinically significant, preventing misdiagnosis as bacterial cystitis and guiding management using established OAB frameworks. The case underscores the importance of considering genitourinary sequelae of COVID-19 and contributes to the growing discussion on long COVID and bladder health.

## Introduction

COVID-19 is widely recognized as a systemic disease with pulmonary, cardiovascular, and neurologic manifestations, yet growing evidence highlights the genitourinary tract as another important site of involvement. A distinct clinical entity, COVID-19-associated cystitis (CAC), has been described in which patients develop de novo or worsening lower urinary tract symptoms (LUTS), such as urgency, frequency, and nocturia without bacterial infection [1]. This case report describes a 71-year-old woman who developed persistent urinary urgency following recovery from COVID-19, successfully treated with oxybutynin. The case is contextualized within the expanding literature on CAC, including evidence from biomarker studies, mechanistic analyses, and large cohort investigations [2]. Emerging data support a pathophysiologic role for both inflammatory cytokine release and viral receptor expression in the bladder, with symptom overlap between CAC, overactive bladder (OAB), and interstitial cystitis [2,3]. Recognition of CAC is clinically significant, preventing misdiagnosis as bacterial cystitis and guiding management using established OAB frameworks. The case underscores the importance of considering genitourinary sequelae of COVID-19 and contributes to the growing discussion on long COVID and bladder health.

## Case presentation

A 71-year-old woman with a medical history of hypertension and type 2 diabetes mellitus developed acute fever, cough, and fatigue. In August of 2025, she presented to the emergency department, and a nasopharyngeal swab confirmed SARS-CoV-2 infection by PCR. She was prescribed Paxlovid was sent home with instructions to return if her symptoms persisted or worsened. The patient reported that a complete reduction in her systemic symptoms occurred within one week. Nine days after diagnosis, she noted urinary urgency, describing a compelling and sudden need to void every one to two hours during the day and waking twice nightly to urinate. Her baseline voiding habits were described as urinating every three to four hours during the daytime and rarely waking at night to void. She denied dysuria, suprapubic pain, hematuria, flank discomfort, or fever. She had no recent history of urinary tract infections or history of overactive bladder or pelvic surgery.

On evaluation nine days after diagnosis at a Manatee County Rural (MCR) Health outpatient clinic, she was afebrile with normal vital signs. Abdominal and pelvic examinations were unremarkable. Urinalysis was negative for leukocyte esterase, nitrites, and microscopic hematuria (Table 1). Urine culture revealed no bacterial growth. Serum creatinine and electrolytes were within normal limits.

**Table 1:** Patient in-office urinalysis

Parameter	Result	Reference Range/Normal
Color	Yellow	Pale yellow to amber
Appearance	Clear	Clear
Specific Gravity	1.02	1.005–1.030
pH	5.6	4.5–8.0
Glucose	Negative	Negative
Protein	Negative	Negative or trace (<30 mg/dL on dipstick)
Ketones	Negative	Negative
Bilirubin	Negative	Negative
Nitrite	Negative	Negative
Leukocyte Esterase	Negative	Negative
Blood	Negative	Negative
RBCs (microscopy)	0–2 /HPF	0–3 /HPF
WBCs (microscopy)	0–2 /HPF	0–5 /HPF
Epithelial Cells	0–5 /HPF	0–5 /HPF
Bacteria	None	None

Given the absence of infection, close temporal relationship to recent COVID-19, and absence of prior urinary symptoms with acute onset, a diagnosis of COVID-19-associated cystitis was made. After discussing conservative management options, she was started on extended-release oxybutynin 10 mg daily due to the bothersome nature of her urgency. Oxybutynin was chosen instead of a β3-agonist because the patient preferred a single daily medication and because she had no contraindications to anticholinergics. At two-week follow-up, she reported marked improvement, and by one month, her symptoms had completely resolved. She discontinued oxybutynin without recurrence of urgency at later follow-up visits at months one and three post diagnosis.

## Discussion

This patient’s course highlights an increasingly recognized clinical syndrome in which SARS-CoV-2 infection is followed by de novo lower urinary tract symptoms in the absence of infection or obstruction. While the symptom pattern of urgency, frequency, and nocturia meets the diagnostic criteria for OAB, several clinical features favor a post-infectious, transient inflammatory process rather than idiopathic OAB. These include her previously normal voiding habits, the abrupt symptom onset after viral recovery, and the complete resolution without recurrence.

Initial reports early in the pandemic noted increased urinary frequency in hospitalized men with COVID-19, despite negative urine cultures and preserved renal function [1]. Subsequent studies identified urinary urgency, frequency, and nocturia in discharged patients, with symptoms persisting for weeks [2]. These clinical findings were reinforced by biomarker analyses demonstrating elevated inflammatory cytokines in the urine of patients with COVID-19 and urinary symptoms, including interleukin-6 (IL-6), interleukin-8 (IL-8), interferon gamma-induced protein 10 (IP-10), and growth-regulated oncogene/C-X-C motif chemokine ligand 1 (GRO/CXCL-1) [1,4]. Importantly, these abnormalities occurred in the absence of detectable bacterial infection, strengthening the case for a unique inflammatory bladder condition associated with COVID-19.

The epidemiology of CAC is now supported by emerging research. A cohort of nearly 1,900 healthcare workers found that individuals with confirmed COVID-19 were two to three times more likely to report new or worsening OAB symptoms compared with uninfected controls [5]. In this study, 22% of symptomatic patients developed de novo OAB within two months of infection, and nearly 38% reported ongoing symptoms at one year [5]. Other follow-up surveys demonstrated that 71% of hospitalized survivors developed new urinary urgency, frequency, or nocturia within 14 weeks of discharge, with nocturia emerging as the most bothersome and quality-of-life-limiting symptom [2]. These findings suggest that urinary complications of COVID-19 are not only common but may persist, aligning with the syndrome of long COVID.

The pathophysiology of CAC is complex and likely multifactorial. One leading mechanism involves cytokine-driven inflammation. Urinary cytokine studies consistently demonstrate elevated levels of IL-6, IL-8, IP-10, GRO/CXCL-1, and C-reactive protein (CRP) in patients with CAC [4,6]. These mediators are known to sensitize bladder afferent pathways, promote detrusor overactivity, and disrupt urothelial barrier function [7]. Inflammatory cytokines such as monocyte chemoattractant protein-1 (MCP-1), C-X-C motif chemokine ligand 10, and regulated upon activation, normal T-cell expressed and secreted (RANTES) are further implicated in bladder dysfunction, with mast cell activation playing a role in perpetuating chronic symptoms [7,8]. The urothelial barrier itself may be compromised, exposing submucosal afferents and amplifying urgency and frequency. A complementary theory involves viral receptor-mediated entry. Angiotensin-converting enzyme 2 (ACE2), transmembrane protease, serine 2 (TMPRSS2), and cluster of differentiation 147 (CD147), the key proteins enabling SARS-CoV-2 cellular entry, are expressed in bladder urothelial and prostate epithelial cells [3]. However, these findings are associative and do not establish that SARS-CoV-2 directly infects or injures urothelial tissue. The presence of viral receptors and urinary cytokines may simply reflect systemic immune activation rather than local viral invasion.

The overlap between CAC, OAB, and interstitial cystitis (IC) is clinically important. OAB is defined by urinary urgency, frequency, and nocturia, with or without urge incontinence, and is typically managed using a tiered strategy of behavioral modification, pharmacologic therapy, and advanced interventions [9]. IC is characterized by chronic inflammation, mast cell activation, and urothelial dysfunction, with elevated urinary cytokines such as IL-6 and CXCL-10 mirroring those seen in CAC [7]. However, IC is distinguished by chronic pelvic or suprapubic pain, which this patient consistently denied. This strongly suggests that CAC represents a non-painful irritative bladder syndrome rather than an IC-like pain disorder.

For clinicians, distinguishing CAC from bacterial cystitis is critical. Patients presenting with urinary urgency or frequency following recent COVID-19 should undergo urinalysis and urine culture, with recognition that most cases will demonstrate sterile urine [1,4]. Awareness of CAC helps avoid unnecessary antibiotic prescriptions, which carry risks of resistance and adverse effects. Once infection has been excluded, management may follow OAB treatment regimens. First-line measures include behavioral modifications such as fluid management, bladder retraining, and pelvic floor exercises [10]. Second-line therapies involve pharmacologic agents, including anticholinergics and β3-adrenergic agonists, which relax the detrusor muscle and reduce urgency [9,10]. Our patient responded well to oxybutynin, consistent with this framework. In refractory cases, neuromodulation or intravesical botulinum toxin injections may be considered, although their role in CAC remains untested [11].

## Conclusions

This case illustrates CAC in a 71-year-old woman who developed persistent urinary urgency following resolution of systemic COVID-19 symptoms. She presented with daytime urinary urgency and nocturia without dysuria, hematuria, or other urinary complaints, and urinalysis and culture were negative. Extended-release oxybutynin was selected based on patient preference, safety considerations, and the need for steady therapeutic levels. Treatment resulted in rapid improvement within two weeks and complete symptom resolution by one month, with sustained remission at three months. This clinical course highlights that CAC can manifest as a de novo lower urinary tract syndrome in the absence of infection or obstruction and that established overactive bladder therapies may be effectively repurposed for symptom management. Recognition of CAC is critical to prevent misdiagnosis, avoid unnecessary antibiotic use, and provide timely symptom relief, particularly in post-COVID patients presenting with new-onset urgency or frequency.

Future research is needed to clarify the epidemiology and natural history of CAC, including risk factors for persistence versus resolution of symptoms. Mechanistic studies investigating inflammatory pathways, cytokine profiles, and potential viral interactions with the bladder urothelium will help elucidate pathophysiology. Clinical trials evaluating pharmacologic, behavioral, and advanced management strategies are warranted to guide evidence-based treatment. Identification of urinary biomarkers may improve diagnosis, monitoring, and risk stratification. Systematic study and increased awareness of CAC will enhance post-COVID patient care, inform management strategies, and help mitigate long-term genitourinary sequelae associated with SARS-CoV-2 infection.
